# A Associação da Relação Proteína C-Reativa/Albumina em Pacientes com Ectasia da Artéria Coronária Isolada

**DOI:** 10.36660/abc.20190476

**Published:** 2020-12-07

**Authors:** Alper Sercelik, Okan Tanrıverdi, Lutfu Askin, Serdar Turkmen

**Affiliations:** 1 Sanko University Faculty of Medicine Gaziantep Turquia Sanko University Faculty of Medicine,Gaziantep - Turquia; 2 Adiyaman University Education and Research Hospital AdıyamanCentury Turquia Adiyaman University Education and Research Hospital - Cardiology,Adıyaman, Century - Turquia

**Keywords:** Proteína C-Reativa, Albuminas, Doença da Artéria Coronariana, Inflamação, Aneurisma Coronário, Dilatação Patológica (ectasia

## Abstract

**Fundamento:**

A ectasia da artéria coronária (EAC) é definida como a dilatação difusa ou localizada do lúmen da artéria coronária com diâmetro de 1,5 a 2,0 vezes o diâmetro da artéria coronária normal adjacente. A relação proteína C-reativa/albumina (CAR, sigla em inglês) é um marcador inflamatório útil que tem sido documentado em doença arterial coronariana.

**Objetivo:**

Analisar a associação entre a EAC e a CAR.

**Métodos:**

Um protocolo caso-controle foi utilizado neste estudo. Foram incluídos 102 pacientesconsecutivos com EAC isolada sem estenose (56 homens e 46 mulheres; idade média de 60,4 ± 8,8 anos). O grupo controle era constituido pelo mesmo número de pacientes pareados por sexo e idade com artérias coronárias normais (55 homens e 47 mulheres; idade média de 61,2 ± 9,1 anos). Características clínicas, achados laboratoriais e histórico de uso de medicamentos foram registrados. Foram realizados teste t de Student, teste U de Mann-Whitney, teste do qui-quadrado, análise de regressão linear e logística. Foi considerado estatisticamente significativo p bilateral < 0,05.

**Resultados:**

A CAR estava aumentada nos pacientes com EAC em comparação com os controles (32 e 16; p < 0,001). Além disso, foi verificado que a CAR era um preditor independente da EAC (razão de chances = 2,202; intervalo de confiança 95%, 1,184 – 5,365; p < 0,001).

**Conclusão:**

No presente estudo, determinamos que os níveis da CAR estavam significativamente mais altos no grupo EAC que no grupo controle e a CAR estava significativamente correlacionada com a EAC. (Arq Bras Cardiol. 2020; [online].ahead print, PP.0-0)

## Introdução

A ectasia da artéria coronária (EAC) é definida como a dilatação difusa ou localizada do lúmen da artéria coronária com diâmetro de 1,5 a 2,0 vezes o diâmetro da artéria coronária normal adjacente. Os aneurismas coronários são definidos como a dilatação luminal com um aumento > 2,0 vezes.^1^ Com o rápido aumento nas aplicações de angiografia coronária (AC), um número crescente de EAC tem sido detectado. Tem sido demonstrado que a EAC é um preditor de mortalidade. As taxas de mortalidade de pacientes com EAC são semelhantes às de pacientes com doença aneurismática não obstrutiva ou doença de três vasos.^2^ A etiopatogênese dessa entidade clínica não é totalmente compreendida. A causa mais comum da EAC na população ocidental é a doença arterial coronariana (DAC) aterosclerótica. As anomalias congênitas são a segunda causa etiológica mais comum. Doença de Kawasaki, doenças do tecido colágeno e doenças do tecido conjuntivo são outras causas comuns da EAC. Os procedimentos invasivos coronários percutâneos e o trauma raramente levam à EAC.^3 , 4^ A dor precordial é geralmente o sintoma primário da EAC. No entanto, arritmia, síndrome coronariana aguda e morte cardíaca súbita são outras condições clínicas observadas na EAC.^5 , 6^

Estudos anteriores demonstraram que a inflamação pode desempenhar um papel na EAC.^7^ A PCR e a albumina têm sido associadas à gravidade da DAC e à presença de complicações cardiovasculares.^8^ A PCR, que é um dos biomarcadores inflamatórios mais amplamente usados, está associada à disfunção endotelial, estado protrombótico, remodelamento e desestabilização das placas ateroscleróticas. Além disso, tem sido verificado que os níveis elevados de PCR em pacientes com carga aterosclerótica estão associados a eventos cardiovasculares significativos.^11^ De outra forma, a inflamação causa a hipoalbuminemia com interrupção do equilíbrio síntese-catabolismo da albumina. A albumina sérica é a proteína sérica mais importante, com funções vitais no corpo humano e possui propriedades antiaterogênicas, incluindo atividades antioxidantes, inibição da ativação plaquetária e modulação e agregação do metabolismo do ácido araquidônico.^15^ Vários estudos anteriores relataram que a hipoalbuminemia está associada ao infarto do miocárdio mais frequente e ao aumento da mortalidade em pacientes com síndrome coronariana aguda.^10 , 16 , 17^ Em comparação com a PCR ou a albumina isoladamente, a relação PCR/albumina (CAR, sigla em inglês), um novo índice de risco baseado na inflamação, demonstrou refletir melhor o prognóstico em pacientes com doença aguda e malignidade.^18^ No entanto, a relação entre a CAR e a EAC ainda não é conhecida. A EAC é uma doença inflamatória; portanto, formulamos a hipótese de que o CAR poderia estar associado ao EAC. Nosso objetivo foi o de investigar a associação entre a EAC e a CAR.

## Métodos

Foi realizado um estudo caso-controle aprovado pelo Comitê de Ética do Hospital Universitário de Sanko. Foram incluídos pacientes com suspeita de isquemia coronária e dor precordial típica após resultados positivos ou equivalentes de testes isquêmicos não invasivos. Todos os pacientes foram submetidos à AC. Durante a AC, os dados digitais de todos os pacientes foram analisados e foram realizadas medidas coronárias quantitativas. O diâmetro do cateter foi utilizado como referência para determinar o diâmetro real do lúmen da artéria coronária. A definição do segmento ectático foi determinada pela realização de pelo menos duas medições nos segmentos proximal, médio e distal das artérias coronárias em pacientes com AC normal e em pacientes que foram considerados como tendo um segmento coronário ectático. A EAC foi definida como a dilatação difusa ou localizada do lúmen da artéria coronária com diâmetro 1,5 a 2,0 vezes o diâmetro da artéria coronária normal adjacente e esses pacientes foram incluídos no grupo EAC isolada. Os pacientes sem placa coronariana ou ectasia foram incluídos no grupo coronária normal.

O histórico médico da população do estudo foi obtido de prontuários e registrado em formulários elaborados para cada paciente. A hipertensão (HT) foi diagnosticada quando a PAS era > 140 mmHg e/ou quando a PAD era > 90 mmHg, ou pelo uso de medicamentos anti-hipertensivos. Diabetes mellitus (DM) foi diagnosticada quando a glicemia de jejum era ≥ 126 mg/dL ou pelo uso de medicamentos antidiabéticos. A hiperlipidemia (HL) foi definida como colesterol total > 200 mg/dL, histórico de dislipidemia e/ou uso de medicamentos antilipidêmicos. Os pacientes que fumavam durante 1 ano ou mais foram definidos como fumantes. O IMC foi determinado usando a fórmula padrão. A FEVE foi calculada automaticamente de acordo com o método de Simpson modificado, com auxílio de um software do aparelho de ecocardiografia.^20^

### Medições laboratoriais

Os níveis de glicose no sangue, creatinina, albumina e PCR foram determinados conforme descrito. A proteína total sérica e a albumina foram medidas por bromine cresol technique usando um analisador C8000 (Abbott Laboratories, IL, EUA). A PCR foi medida por nefelometria (BN ProSpec System, Siemens). A taxa de filtração glomerular estimada foi determinada utilizando a equação Cockcroft-Gault.

### Angiografia coronária

Foi realizada a AC pelo método de Judkins, via femoral, utilizando os ângulos cranial e caudal nos planos inclinados direito e esquerdo a 30 fps. As imagens de AC dos pacientes foram analisadas por cardiologistas intervencionistas cegos ao estudo. A EAC já foi definida por Falsetti e Carroll;^21^ o nosso estudo utilizou o mesmo método. Os segmentos normais foram definidos como a ausência de estenose ou ectasia determinada pela AC. Os casos de EAC com estenose coronariana foram excluídos do estudo.

### Análise estatística

A análise estatística foi realizada utilizando SPSS v25 (SPSS Inc., EUA). A normalidade das variáveis contínuas foi testada pelo teste de Kolmogorov-Smirnov e apresentada como média e desvio padrão ou mediana e intervalo interquartil, de acordo com a normalidade dos dados. As variáveis contínuas com distribuição normal foram comparadas pelo teste t de Student e o teste U de Mann-Whitney foi usado para aquelas com distribuição não normal. Foi utilizado o teste t de Student para valores não pareados. Os dados categóricos foram comparados usando o teste do qui-quadrado. Na análise de regressão linear univariada, as variáveis com nível de significância p < 0,25 foram definidas como potenciais marcadores de risco e incluídas como variáveis comuns em todo o modelo de variável. A análise de regressão logística foi realizada para obter determinantes independentes da EAC. Foi considerado estatisticamente significativo p bilateral < 0,05.

## Resultados

Dos 226 pacientes, 8 foram excluídos por causa de infarto do miocárdio e disfunção ventricular esquerda; 5 foram excluídos por hipertrofia ventricular esquerda e doença valvar cardíaca e 6 foram excluídos por HT e insuficiência renal (n = 6). Além disso, 3 pacientes foram excluídos por outros motivos, como doença cerebrovascular, disfunção hepática, doença autoimune, doença neoplásica e osteoporose (n = 3). Após essas exclusões, 204 pacientes foram inscritos. Foram incluídos 102 pacientes com EAC isolada sem estenose da artéria coronária (56 homens e 46 mulheres; idade média 60,4 ± 8,8 anos) no grupo paciente; o grupo controle foi constituído pelo mesmo número de indivíduos consecutivos com artérias coronárias angiograficamente normais (55 homens e 47 mulheres; idade média 61,2 ± 9,1 anos).

A Tabela 1 mostra os dados dos pacientes. As características demográficas mostraram grupos pareados por idade e sexo. Fatores de risco de DAC como DM também foram semelhantes, mas outros fatores de risco (tabagismo, HT, HL e histórico familiar) foram significativamente maiores no grupo EAC do que no grupo controle (p < 0,001, p < 0,001, p = 0,006 e p = 0,022, respectivamente). Nenhuma mudança foi observada em termos de regimes de tratamento.

**Tabela 1:** – Características de linha de base da população do estudo

Variáveis	Grupo EAC (n = 102)	Grupo controle (n = 102)	Valor p
Idade, anos	60,4 ± 8,8	61,2 ± 9,1	0,422
Sexo (masculino), n, (% )	56 (54,9)	55 (53,9)	0,740
IMC (kg/m^2^)	27,4±3,1	25,2± 3,2	0,317
Diabetes mellitus, n (%)	15 (14,7)	12 (11,7)	0,422
Tabagismo, n, (%)	40 (39,2)	18 (17,6)	< 0,001
Hipertensão, n (%)	38 (37,2)	27 (26,4)	< 0,001
Hiperlipidemia, n (%)	22 (21,5 )	10 (9,8)	0,006
Histórico familiar, n, (%)	15 (14,7)	8 (7,8)	0,022
**Medicamentos prévios, n, (%)**			
Ácido acetilsalicílico	20 (19,6)	16 (15,6)	0,224
Betabloqueadores	22 (21,5)	17 (16,6)	0,314
IECA/BRA	17 (16,6)	13 (12,7)	0,509
Estatinas	10 (9,8)	7 (7,1)	0,356
FEVE, (%)	60,2±3,4	61,3±3,9	0,533
PAS (mmHg)	122,0±9,1	118,6±7,5	0,424
PAD (mmHg)	88,0±7,2	85,6±4,1	0,358
Frequência cardíaca (batidas/m)	75,2±9	73,8±7	0,411
Hemoglobina, g/dL	13,1+1,8	12,7+1,7	0,388
Contagem de leucócitos, 10^3^/mL	8,6±2,9	8,4±2,2	0,758
Contagem de plaquetas, 10^3^/mL	232,7±77,4	244,8±75,2	0,554
Glicemia de jejum (mg/dL)	127,1±42,8	123,1±45,2	0,146
Creatinina, mg/dL	0,86 (0,75-0,99)	0,85 (0,75-0,97)	0,785
TFGe, mL/min	92,3 (68,9-105,6)	93,8 (74,9-107,9)	0,656
Colesterol total, mg/dL	168,0±36,0	158,2±39,8	0,411
HDL (mg/dL)	33,6±7,0	39,8±8,0	0,012
LDL (mg/dL)	123,2±32,6	98,4±29,4	< 0,001
Triglicerídeos, mg/dL	129 + 61	122 + 58	0,188
Proteína C-reativa, mg/dL	1,25 (0,50-2,84)	0,62 (0,27-1,16)	< 0,001
Albumina, g/dL	3,76 + 0,42	4,02 + 0,32	< 0,001
CAR, *100	32 (12-68)	16 (6-30)	< 0,001

*BRA: bloqueadores dos receptores da angiotensina; CAR: relação proteína C-reativa/albumina; FEVE: fração de ejeção do ventrículo esquerdo; HDL: lipoproteína de alta densidade; IECA: inibidores da enzima conversora de angiotensina; IMC: índice de massa corporal; LDL: lipoproteína de baixa densidade; PAD: pressão arterial diastólica; PAS: pressão arterial sistólica; TFGe: taxa de filtração glomerular estimada.*

A avaliação de IMC, PAS, PAD, FEVE, frequência cardíaca e glicemia de jejum não revelou diferenças significativas. Os parâmetros do painel lipídico, triglicerídeos e grupos de colesterol total foram semelhantes; o HDL foi mais alto nos controles (p = 0,012) e o LDL foi maior nos pacientes com EAC (p < 0,001). A PCR, a albumina e a CAR diferiram significativamente entre os grupos (p < 0,001). Houve semelhança entre os grupos em relação aos outros parâmetros laboratoriais.

Aplicando um modelo de regressão logística univariada, DM, tabagismo, HT e CAR correlacionaram-se com a EAC. A análise de regressão revelou que tabagismo, HT e CAR foram preditores independentes de EAC (tabagismo: razão de chances [RC] 1,812 [IC 95% 1,124 – 2,655; p = 0,024], HT: RC 2,175 [IC 95% 1,156 – 4,227; p < 0,001], CAR: RC 2,202 [IC 95% 1,184 – 5,365; p < 0,001]) ( Tabela 2 ).

**Tabela 2:** – Fatores associados com a ectasia da artéria coronária

Análise de regressão linear				Análise de regressão logística		
	Coeficientes	IC 95%	Valor p	RC	IC 95%	Valor p
Idade, anos	0,052	0,013-0,107				
FEVE	0,002	−0,018-0,026				
IMC (kg/m^2^)	0,030	0,010-0,073				
Diabetes mellitus	0,168	0,011- 0,524	0,024*	1,277	0,811-1,613	0,102
Tabagismo	0,322	0,010-1,114	0,007*	1,812	1,124-2,655	0,024*
Hipertensão	0,533	0,017-1,010	0,003*	2,175	1,156-4,227	< 0,001*
Hiperlipidemia	0,025	−0,020-0,056				
PAS (mmHg)	0,068	−0,017-0,122				
PAD (mmHg)	0,024	−0,002-0,048				
Frequência cardíaca (batidas/m)	0,074	−0,024-0,172				
CAR, *100	0,618	0,119-1,496	< 0,001*	2,202	1,184-5,365	< 0,001*
HDL (mg/dL)	−0,076	−0,312-0,025				
LDL (mg/dL)	0,009	−0,057-0,020				

*Valor p < 0,05. As variáveis com p < 0,25 na regressão univariada foram incluídas na regressão multivariada. CAR: relação proteína C-reativa/albumina; FEVE: fração de ejeção do ventrículo esquerdo; HDL: lipoproteína de alta densidade; IMC: índice de massa corporal; LDL: lipoproteína de baixa densidade; PAD: pressão arterial diastólica; PAS: pressão arterial sistólica.

## Discussão

Este relato mostra que a CAR foi significativamente mais alta no grupo EAC do que no grupo controle. Até onde sabemos, somos os primeiros a mostrar que a CAR está intimamente associada à EAC.

Ainda não está claro quais fatores locais ou globais estão envolvidos na patogênese da EAC. Foi relatado que a EAC é causada pela anormalidade generalizada na parede vascular contendo vários segmentos e que representa ectasia sacular em vez de ectasia fusiforme.^22^ Um estudo relativamente limitado sobre o prognóstico foi realizado para pacientes com EAC. Trinta anos atrás, o maior estudo de coorte de EAC descobriu que os pacientes aneurismáticos tinham uma taxa de mortalidade em 5 anos de 26%.^23^ Kajinami et al.,^26^ examinaram a autópsia de um paciente com EAC e hipercolesterolemia familiar, que morreu no século XX devido a um infarto agudo do miocárdio. O exame microscópico revelou uma grande quantidade de células plasmáticas, macrófagos e infiltração de linfócitos nas camadas íntima/média das artérias coronárias. Durante o exame patológico da EAC, foram observadas evidências de reações ateroscleróticas, como hialinização comum típica, calcificação focal e fibrose, acúmulo de lipídios, dano intimal e medial, colesterol, hemorragia e corpo estranho de células gigantes.

Outro fator potencial que leva ao desenvolvimento da EAC é o óxido nítrico (ON), que pode causar dilatação coronária devido à hiperestimulação do endotélio. Muitos pacientes têm recebido o trinitrato de gliceril crônico para angina, que pode piorar a ectasia por meio da estimulação do ON. Esses pacientes podem ter DAC e a aterosclerose demonstrou causar liberação inadequada de ON endotelial.^25^ Quyyumi et al.,^26^ demonstraram a relação entre o ON e a aterosclerose relatando que a dilatação vascular coronária foi causada pelo aumento do ON, devido à acetilcolina sem aterosclerose angiograficamente comprovada.

O mecanismo patológico subjacente da EAC ainda não está totalmente compreendido. Embora não tenha sido confirmada uma relação definitiva entre a aterosclerose e a EAC, a EAC é considerada uma variante de DAC e a principal causa de EAC é a aterosclerose.^23 , 27^ O papel da inflamação no processo da aterosclerose é bem documentado.^28^ A aterosclerose está associada à formação de aneurisma que se estende até a túnica média durante um processo inflamatório, que termina com a degeneração da média cística. ^31^ Estudos anteriores demonstraram que marcadores inflamatórios, como moléculas de adesão solúveis no plasma, leucócitos, adiponectina, fosfolipase-A2 associada à lipoproteína, PCR, inibidor do ativador do plasminogênio-1, IL-1, TNF-alfa e IL-10 foram significativamente aumentados em pacients com EAC.^32^

Vários estudos anteriores mostraram que a CAR está associada à aterosclerose e sugeriram que deveria ser considerada um marcador de risco cardiovascular. Este estudo verificou que a CAR foi significativamente mais alta em pacientes com EAC do que no grupo controle e apoia a hipótese de que a aterosclerose causa EAC.

As células isquêmicas ou necróticas danificadas causam uma resposta inflamatória sistêmica ao liberar agentes pró-inflamatórios no tecido e no plasma. O prognóstico da doença pode mudar com a velocidade da inflamação.^33^ Tem sido demonstrado que a aterosclerose demonstrou está fortemente correlacionada com o aumento da PCR sérica.^34^ Além disso, tem sido mostrado que a PCR está associada com disfunção coronária endotelial dependente e independente em pacientes com DAC,^35^ sugerindo que o aumento da PCR pode predizer a disfunção em pacientes com STEMI e pode ser um forte preditor do fenômeno de não refluxo ( *no-reflow* ).^36^ Em nosso estudo, os níveis elevados da PCR demonstrarm uma forte associação entre a EAC e a PCR.

A hipoalbuminemia não é apenas um fator de risco; também indica mau prognóstico em pacientes com STEMI.^37 , 38^ Foi documentado que o aumento da inflamação contribui para a síntese e a degradação da albumina.^39^ A hipoalbuminemia leva a muitas complicações, incluindo a disfunção endotelial, bem como a agregação plaquetária e a estenose da artéria coronária induzida por disfunção plaquetária.^40^ Em um estudo de 1.303 indivíduos com síndrome coronariana aguda, foi verificado que os níveis séricos de albumina estavam associadas à gravidade da DAC.^10^ Em nosso estudo, houve uma correlação negativa entre o nível sérico de albumina level e a EAC.

Acredita-se que a CAR, conforme originalmente descrita por Fairclough et al.,^42^ seja melhor do que a PCR e a albumina isoladamente para predizer complicações médicas.^42^ A inflamação é uma das características da aterogênese e a CAR demonstra as condições inflamatórias.

A CAR foi recentemente investigada como um biomarcador potencial para prever as consequências de eventos cardiovasculares adversos.^43^ Cagdas et al.,^44^ mostraram que a CAR e a gravidade da DAC estavam associadas. Em pacientes com câncer maligno, a CAR previu o prognóstico e a progressão da doença.^45 , 46^ Por isso, a CAR é um biomarcador mais confiável para a previsão da gravidade da doença. Relatórios anteriores que avaliaram a CAR na DAC mostraram resultados promissores. Um estudo de STEMI mostrou que a contagem de leucócitos, a relação neutrófilos/linfócitos e a CAR se correlacionaram com o fenômeno de não refluxo.^43^

Os resultados do nosso estudo mostraram que a associação da CAR com a EAC foi significativa. Este é o primeiro estudo a mostrar uma associação entre a EAC e os níveis mais elevados da CAR. O aumento da CAR foi um marcador prognóstico da EAC. Os resultados de um estudo sobre a relação entre a hipercolesterolemia familiar e a EAC mostraram que a dislipidemia foi uma das causas da EAC.^47^ Em nosso estudo, foram observados níveis elevados de LDL e baixos de HDL em pacientes com EAC. Nenhuma mudança significativa foi encontrada nos níveis de triglicerídeos em pacientes com EAC em comparação com os controles. Uma forte relação foi demonstrada entre HT e EAC.^48^ Em nosso estudo, a prevalência de HT foi maior entre pacientes com EAC e a HT foi independentemente associada com a EAC.

Observamos que a CAR foi mais alta nos pacientes do que no grupo controle. Supomos que a CAR elevada pode predizer o risco de aterosclerose em pacientes com EAC. Uma revisão da literatura mostra que a EAC não é uma condição clínica inocente e que estudos maiores serão necessários no futuro para criar a melhor estratégia de tratamento e gerenciamento de risco.

### Limitações do estudo

Estudos mais abrangentes e multicêntricos são necessários para melhor explicar a variabilidade dos marcadores inflamatórios e o papel preditor dos níveis séricos da CAR. A significância prognóstica da CAR não foi avaliada e deverá ser estabelecida em investigações futuras. Este foi um estudo caso-controle e, portanto, não foi possível obter dados de mortalidade. Embora a CAR seja aceita como um novo marcador miocárdico sensível, sua especificidade na determinação da presença de EAC tem sido questionada, porque muitas outras condições, especialmente outras infecções, também podem aumentar os níveis da CAR. Os níveis da CAR podem ser afetados por outros fatores importantes, como idade, sexo e raça. Finalmente, em vez de usar métodos quantitativos, como ultrassom intravascular, a avaliação visual foi o único método usado para diagnosticar e excluir pacientes.

## Conclusão

Nosso estudo demostra que os níveis da CAR são mais altos nos pacientes com EAC em comparação aos pacientes com artérias coronárias normais. Os níveis altos da CAR podem suportar a hipótese de que a CAR possa estar relacionada ao desenvolvimento da EAC. Em nosso estudo, os altos níveis da CAR estavam significativamente correlacionados com a EAC.

## References

[1] 1. Aktürk E, Aşkın L, Nacar H, Taşolar MH, Türkmen S, Çetin M, et al. Association of serum prolidase activity in patients with isolated coronary artery ectasia. Anatol J Cardiol. 2018;19(2):110-6. 10.14744/AnatolJCardiol.2017.8160 PMC5864804 29339675

[2] 2. Baman TS, Cole JH, Devireddy CM, Sperling LS. Risk factors and outcomes in patients with coronary artery aneurysms. Am J Cardiol. 2004;93(12):1549-51. 10.1016/j.amjcard.2004.03.011 15194034

[3] 3. Sayin T, Döven O, Berkalp B, Akyürek O, Güleç S, Oral D. Exercise induced myocardial ischemia in patients with coronary artery ectasia without obstructive coronary artery disease. Int J Cardiol. 2001;78(2):143-9. 10.1016/s0167-5273(01)00365-5 11334658

[4] 4. Maehara A, Mintz GS, Ahmed JM, Fuchs S, Castagna MT, Pichard AD, et al. An intravascular ultrasound classification of angiographic coronary artery aneurysms. Am J Cardiol. 2001;88(4):365-70. 10.1016/s0002-9149(01)01680-0 11545755

[5] 5. Mrdovic I, Jozic T, Asanin M, Perunicic J, Ostojic M. Myocardial reinfarction in a patient with coronary ectasia. Cardiology. 2004;102(1):32-4. 10.1159/000077000 15004454

[6] 6. Rosenberg VD, Nepomnyashchikh LM. Pathomorphological peculiarities of coronary artery ectasias and their role in the pathogenesis of sudden cardiac death. Bull Exp Biol Med. 2004;138(5):515-21. 10.1007/s10517-005-0085-9 15723141

[7] 7. Ozcan OU, Gulec S. Coronary artery ectasia. Cor Vasa. 2013;55(3):242-7.

[8] 8. Bisoendial RJ, Boekholdt SM, Vergeer M, Stroes ES, Kastelein JJ. C-reactive protein is a mediator of cardiovascular disease. Eur Heart J. 2010;31(17):2087-91. 10.1093/eurheartj/ehq238 20685682

[9] 9. Karadeniz M, Duran M, Akyel A, Yarlıoğlueş M, Öcek AH, Çelik İE, et al. High sensitive CRP level is associated with intermediate and high SYNTAX score in patients with acute coronary syndrome. Int Heart J. 2015;56(4):377-80. 10.1536/ihj.14-299 26118590

[10] 10. Kurtul A, Murat SN, Yarlioglues M, Duran M, Ocek AH, Koseoglu C, et al. Usefulness of serum albumin concentration to predict high coronary SYNTAX score and in-hospital mortality in patients with acute coronary syndrome. Angiology. 2016;67(1):34-40. 10.1177/0003319715575220 25783433

[11] 11. Bisoendial RJ, Kastelein JJ, Peters SL, Levels JH, Birjmohun R, Rotmans JI, et al. Effects of CRP infusion on endothelial function and coagulation in normocholesterolemic and hypercholesterolemic subjects. J Lipid Res. 2007;48(4):952–60. 10.1194/jlr.P600014-JLR200 17259597

[12] 12. Devaraj S, Kumaresan PR, Jialal I. Effect of C-reactive protein on chemokine expression in human aortic endothelial cells. J Mol Cell Cardiol. 2004;36(3):405–10. 10.1016/j.yjmcc.2003.12.005 15010279

[13] 13. Taniguchi H, Momiyama Y, Ohmori R, Yonemura A, Yamashita T, Tamai S, et al. Associations of plasma C-reactive protein levels with the presence and extent of coronary stenosis in patients with stable coronary artery disease. Atherosclerosis. 2005;178(1):173–7. 10.1016/j.atherosclerosis.2004.08.012 15585215

[14] 14. Haverkate F, Thompson SG, Pyke SD, Gallimore JR, Pepys MB. Production of C-reactive protein and risk of coronary events in stable and unstable angina. European Concerted Action on Thrombosis and Disabilities Angina Pectoris Study Group. Lancet. 1997;349(9050):462–6. 10.1016/s0140-6736(96)07591-5 9040576

[15] 15. Purdon AD, Rao AK. Interaction of albumin, arachidonic acid and prostanoids in platelets. Prostaglandins, Leukot Essent Fat Acids. 1989;35(4):213–8. 10.1016/0952-3278(89)90004-5 2654961

[16] 16. Nelson JJ, Liao D, Sharrett AR, Folsom AR, Chambless LE, Shahar E, et al. Serum albumin level as a predictor of incident coronary heart disease: the Atherosclerosis Risk in Communities (ARIC) study. Am J Epidemiol. 2000;151(5):468–77. 10.1093/oxfordjournals.aje.a010232 10707915

[17] 17. Oduncu V, Erkol A, Karabay CY, Kurt M, Akgun T, Bulut M, et al. The prognostic value of serum albumin levels on admission in patients with acute ST-segment elevation myocardial infarction undergoing a primary percutaneous coronary intervention. Coron Artery Dis. 2013;24(2):88–94. 10.1097/MCA.0b013e32835c46fd 23249632

[18] 18. Fairclough E, Cairns E, Hamilton J, Kelly C. Evaluation of a modified early warning system for acute medical admissions and comparison with C-reactive protein/albümin ratio as a predictor of patient outcome. Clin Med (Lond). 2009;9(1):30–3. 10.7861/clinmedicine.9-1-30 PMC5922628 19271597

[19] 19. Kinoshita A, Onoda H, Imai N, Iwaku A, Oishi M, Tanaka K, et al. The C-reactive protein/albumin ratio, a novel inflammation-based prognostic score, predicts outcomes in patients with hepatocellular carcinoma. Ann Surg Oncol. 2015;22(33):803–10. 10.1245/s10434-014-4048-0 25190127

[20] 20. Schiller NB, Acquatella H, Ports TA, Drew D, Goerke J, Ringertz H, et al. Left ventricular volume from paired biplane two-dimensional echocardiography. Circulation. 1979; 60(3):547-55. 10.1161/01.cir.60.3.547 455617

[21] 21. Falsetti HL, Carroll RJ. Coronary artery aneurysm: a review of the literature with a report of 11 new cases. Chest. 1976; 69(5):630-6. 10.1378/chest.69.5.630 1083790

[22] 22. Williams MJ, Stewart RA. Coronary artery ectasia: local pathology or diffuse disease? Cathet Cardiovasc Diagn. 1994; 33(2):116-9. 10.1002/ccd.1810330206 7834723

[23] 23. Swaye PS, Fisher LD, Litwin P, Vignola PA, Judkins MP, Kemp HG, et al. Aneurysmal coronary artery disease. Circulation. 1983;67(1):134-8. 10.1161/01.cir.67.1.134 6847792

[24] 24. Kajinami K, Kasashima S, Oda Y, Koizumi J, Katskuda S, Mabuchi H. Coronary ectasia in familial hypercholesterolemia: histopathologic study regarding matrix metalloproteinases. Mod Pathol. 1999; 12(12):1174-80. 10619272

[25] 25. Kahraman F, Karabacak M, Türker Y. Serum nitric oxide level in patients with coronary artery ectasia. Anatol J Cardiol. 2017; 17(4):341. 10.14744/AnatolJCardiol.2017.7738 PMC5469118 28466831

[26] 26. Quyyumi AA, Dakak N, Andrews NP, Husain S, Arora S, Gilligan DM, et al. Nitric oxide activity in the human coronary circulation. Impact of risk factors for coronary atherosclerosis. J Clin Invest. 1995; 95(4): 1747-55. 10.1172/JCI117852 PMC295695 7706483

[27] 27. Cohen P, O’Gara PT. Coronary artery aneurysms: a review of the natural history, pathophysiology, and management. Cardiol Rev. 2008;16(6):301-4. 10.1097/CRD.0b013e3181852659 18923233

[28] 28. Ozde C, Korkmaz A, Kundi H, Oflar E, Ungan I, Xankisi V, et al. Relationship between plasma levels of soluble CD40 ligand and the presence and severity of isolated coronary artery ectasia. Clin Appl Thromb Hemost. 2018;24(2):379-86. 10.1177/1076029616680476 PMC6714671 27879468

[29] 29. Xu Y, Yu Q, Yang J, Yuan F, Zhong Y, Zhou Z, et al. Acute hemodynamic effects of remote ischemic preconditioning on coronary perfusion pressure and coronary collateral blood flow in coronary heart disease. Acta Cardiol Sin. 2018;34(4):299-306. 10.6515/ACS.201807_34(4).20180317A PMC6066945 30065567

[30] 30. Zhao ZW, Ren YG, Liu J. Low serum adropin levels are associated with coronary slow flow phenomenon. Acta Cardiol Sin. 2018;34(4):307-12. 10.6515/ACS.201807_34(4).20180306B PMC6066951 30065568

[31] 31. Nichols L, Lagana S, Parwani A. Coronary artery aneurysm: a review and hypothesis regarding etiology. Arch Pathology Lab Med 2008;132(5):823-8. 10.5858/2008-132-823-CAAARA 18466032

[32] 32. Turhan H, Erbay AR, Yasar AS, Askoy Y, Bicer A, Yetkin G, et al. Plasma soluble adhesion molecules; intercellular adhesion molecule-1, vascular cell adhesion molecule-1 and E-selectin levels in patients with isolated coronary artery ectasia. Coron Artery Dis. 2005;16(1):45-50 10.1097/00019501-200502000-00009 15654200

[33] 33. Kottoor SJ, Arora RR. The utility of anti-inflammatory agents in cardiovascular disease: a novel perspective on the treatment of atherosclerosis. J Cardiovasc Pharmacol Ther. 2018: 23(6):483-493. 10.1177/1074248418778548 29783850

[34] 34. Yousuf O, Mohanty BD, Martin SS, Joshi PH, Blaha MJ, Nasir K, et al. High-sensitivity C-reactive protein and cardiovascular disease: a resolute belief or an elusive link? J Am Coll Cardiol. 2013;62(5):397-408. 10.1016/j.jacc.2013.05.016 23727085

[35] 35. Tomai F, Ribichini F, Ghini AS, Ferrero V, Andò G, Vassanelli C, et al. Elevated C-reactive protein levels and coronary microvascular dysfunction in patients with coronary artery disease. Eur Heart J. 2005; 26(20):2099-105. 10.1093/eurheartj/ehi356 15961409

[36] 36. Huet F, Akodad M, Kuster N, Kovacsik H, Leclercq F, Dupuy AM, et al. An hs-TNT second peak associated with high CRP at day 2 appears as potential biomarkers of micro-vascular occlusion on magnetic resonance imaging after reperfused ST-segment elevation myocardial infarction. Cardiology. 2018;140(4):227-36. 10.1159/000490881 30138917

[37] 37. Oduncu V, Erkol A, Karabay CY, Kurt M, Akgun T, Bulut M, et al. The prognostic value of serum albumin levels on admission in patients with acute STsegment elevation myocardial infarction undergoing a primary percutaneous coronary intervention. Coron Artery Dis. 2013; 24(2):88-94. 10.1097/MCA.0b013e32835c46fd 23249632

[38] 38. Nelson J, Liao D, Sharrett AR, Folsom AR, Chambless LE, Shahar E, et al. Serum albumin level as a predictor of incident coronary heart disease: the Atherosclerosis Risk in Communities (ARIC) study. Am J Epidemiol. 2000; 151(5):468-77. 10.1093/oxfordjournals.aje.a010232 10707915

[39] 39. Don BR, Kaysen GA. Serum albumin: relationship to inflammation and nutrition. Semin Dial. 2004;17(6):432-7. 10.1111/j.0894-0959.2004.17603.x 15660573

[40] 40. Joles JA, Willekes-Koolschijn N, Koomans HA. Hypoalbuminemia causes high blood viscosity by increasing red cell lysophosphatidylcholine. Kidney Int. 1997;52(3):761-70. 10.1038/ki.1997.393 9291198

[41] 41. Mikhailidis DP, Ganotakis ES. Plasma albumin and platelet function: relevance to atherogenesis and thrombosis. Platelets. 1996; 7(3):125-37. 10.3109/09537109609023571 21043591

[42] 42. Fairclough E, Cairns E, Hamilton J, Kelly C. Evaluation of a modified early warning system for acute medical admissions and comparison with C-reactive protein/albumin ratio as a predictor of patient outcome. Clin Med. 2009;9(1):30-3. 10.7861/clinmedicine.9-1-30 PMC5922628 19271597

[43] 43. Duman H, Çinier G, Bakırcı EM, et al. Relationship Between C-Reactive Protein to Albumin Ratio and Thrombus Burden in Patients With Acute Coronary Syndrome. Clin Appl Thromb Hemost 2019;25:1076029618824418. doi: 10.1177/1076029618824418. 10.1177/1076029618824418 PMC6715111 30808220

[44] 44. Çağdaş M, Rencüzoğullari I, Karakoyun S, Karabag Y, Yesin M, Artaç I, et al. Assessment of Relationship Between C-Reactive Protein to Albumin Ratio and Coronary Artery Disease Severity in Patients With Acute Coronary Syndrome. Angiology. 2019;70(4):361-8. 10.1177/0003319717743325 29172653

[45] 45. Yoshida N, Baba H. The C-reactive protein/albumin ratio may predict the long-term outcome in patients with malignant pleural mesothelioma. Ann Surg Oncol. 2018;25(6): 1471-2. 10.1245/s10434-018-6420-y 29516363

[46] 46. Chen Z, Shao Y, Fan M, Zhuang Q, Wang K, Cao W, et al. Prognostic significance of preoperative C-reactive protein: albumin ratio in patients with clear cell renal cell carcinoma. Int J Clin Exp Pathol. 2015;8(11):14893-900. PMC4713605 26823819

[47] 47. Takajashi K, Ohyanagi M, Ikeoka K, Tateishi J, Iwasaki T. Clinical course of patients with coronary ectasia. Cardiology 1999; 91(3):145-9. 10.1159/000006901 10516406

[48] 48. Yilmaz H, Sayar N, Yilmaz M, Tangürek B, Cakmak N, Gürkan U, et al. Coronary artery ectasia: clinical and angiographical evaluation. Turk Kardiyol Dern Ars 2008; 36(8):530-5. 19223718

