# Supplementary material for: A Associação da Relação Proteína C-Reativa/Albumina em Pacientes com Ectasia da Artéria Coronária Isolada
Source: Arq Bras Cardiol. 2020 Dec 7;116(1):48–54. [Article in Portuguese] doi: 10.36660/abc.20190476 (PMC8159494; doi:10.36660/abc.20190476)
Supplement: Translation

# Translation: Association of C-Reactive Protein to Albumin Ratio in Patients with Isolated Coronary Artery Ectasia

**Authors:** Alper Sercelik, Okan Tanrıverdi, Lutfu Askin, Serdar Turkmen

**Affiliations:** 1 Turkey Sanko University Faculty of Medicine, Cardiology, Gaziantep - Turkey; 2 Turkey Adiyaman University Education and Research Hospital - Cardiology, Adiyaman - Turkey

**Keywords:** C-Reactie Protein, Albumins, Coronary Artery Disease/complications, Inflammation, Coronary Aneurysm, Dilatation, Pathologic (ectasig

## Abstract

**Background:**

Coronary artery ectasia (CAE) is defined as diffuse or localized dilatation of coronary artery lumen with a diameter of 1.5 to 2.0 times the adjacent normal coronary artery. The C-reactive protein to albumin ratio (CAR) is a useful inflammatory marker, which has been documented in coronary artery disease.

**Objective:**

To analyze the association of CAE and CAR.

**Methods:**

A case-control protocol was used in this study. We included 102 consecutive patients with isolated CAE without stenosis (56 men and 46 women; mean age 60.4 ± 8.8 years). The control subjects consisted of an equal number of sex and age matched patients with normal coronary arteries (55 men and 47 women; mean age 61.2 ± 9.1 years). Clinical features, laboratory findings, and medication use history were recorded. Student’s t test, Mann-Whitney U test, chi-square test, and linear and logistic regression analysis were performed. A 2-sided p < 0.05 was statistically considered significant.

**Results:**

The CAR was increased in patients with CAE compared to the controls (32 and 16; p < 0.001). In addition, the CAR was found to be an independent predictor of CAE (OR = 2.202; 95% CI 1.184 – 5.365; p < 0.001).

**Conclusion:**

In the present study, we determined that CAR levels were significantly higher in the CAE group than in the control group, and the CAR was significantly correlated with CAE. (Arq Bras Cardiol. 2020; [online].ahead print, PP.0-0)

## Introduction

Coronary artery ectasia (CAE) is defined as diffuse or localized dilatation of coronary artery lumen with a diameter 1.5 to 2.0 times the adjacent normal coronary artery. Coronary aneurysms are defined as luminal dilatation with a > 2.0 fold increase.^1^ With the rapid increase in applications of coronary angiography (CA), an increasing number of CAE have been detected. CAE has been shown to be a predictor of mortality. The mortality rates of patients with CAE are similar to those of patients with non-obstructive aneurysmal or 3-vessel disease.^2^ The etiopathogenesis of this clinical entity is not fully understood. The most common cause of CAE in the Western population is atherosclerotic coronary artery disease (CAD). Kawasaki disease, collagen tissue diseases, and connective tissue diseases are the other causes of CAE. Percutaneous coronary invasive procedures and trauma rarely lead to CAE.^3 - 4^ Chest pain is usually the primary symptom of CAE. However, arrhythmia, acute coronary syndrome, and sudden cardiac death are other observed clinical conditions of CAE.^5 , 6^

Previous studies have shown that inflammation may play a role in CAE.^7^ CRP and albumin have been linked to CAD severity and to the presence of cardiovascular complications.^8 - 10^ CRP, which is one of the most commonly used inflammatory biomarkers, is associated with endothelial dysfunction, prothrombotic status, remodeling, and destabilization of atherosclerotic plaques. Furthermore, elevated CRP levels in patients with atherosclerotic burden have been found to be associated with significant cardiovascular events.^11 - 14^ On the other hand, inflammation causes hypoalbuminemia with disruption of albumin synthesis-catabolism balance. Serum albumin is the most important serum protein with vital functions in the human body, and it has anti-atherogenic properties, including antioxidant activities, inhibition of platelet activation, and modulation and aggregation of arachidonic acid metabolism.^15^ Several previous studies have reported that hypoalbuminemia is associated with more frequent myocardial infarction and increased mortality in patients with acute coronary syndrome.^10 , 16 , 17^ In comparison with CRP or albumin alone, the CRP to albumin ratio (CAR), a new inflammation-based risk index, has been shown to better reflect prognosis in patients with acute medical condition and malignancy.^18 , 19^ However, the relationship between the CAR and CAE is not yet known. CAE is an inflammatory disease; thus, we hypothesized that the CAR could be associated with CAE. Our aim was to investigate the association between CAE and the CAR.

## Methods

A case-control study approved by the Ethics Committee in Sanko University Hospital was performed. Patients with suspected coronary ischemia and typical chest pain following positive or equivalent results of noninvasive ischemic tests were included. All patients underwent CA. During CA, digital data of all patients were analyzed, and quantitative coronary measurements were performed. Catheter diameter was used as the reference to determine the actual coronary artery lumen diameter. Definition of the ectatic segment was determined by performing at least two measurements at the proximal, middle, and distal segments of the coronary arteries in patients with normal CA and in patients who were considered to have an ectatic coronary segment. CAE was defined as diffuse or localized dilatation of coronary artery lumen with a diameter 1.5 to 2.0 times the adjacent normal coronary artery, and these patients were included in the isolated CAE group. Patients without coronary plaque or ectasia were included in the normal coronary group.

The medical history of the study population was obtained from medical records and recorded in forms prepared for each patient. Hypertension (HT) was diagnosed when SBP was > 140 mmHg and/or when DBP > 90 mmHg, or by antihypertensive drug use. Diabetes mellitus (DM) was diagnosed when fasting blood glucose was ≥ 126 mg/dL or by antidiabetic drug use. Hyperlipidemia (HL) was defined as total cholesterol level > 200 mg/dL, history of dyslipidemia, and/or antilipidemic drug use. Patients who were smokers for 1 year or more were defined as smokers. BMI was determined using the standard formula. The LVEF was automatically calculated according to the modified Simpson’s method, with the help of software on the echocardiography device.^20^

### Laboratory Measurements

Blood glucose, creatinine, albumin, and CRP levels were determined as described. Serum total protein and albumin were measured by bromine cresol technique using a C8000 analyzer (Abbott Laboratories, IL, USA). CRP was measured by nephelometry (BN ProSpec System, Siemens). The estimated glomerular filtration rate was determined using the Cockcroft-Gault equation.

### Coronary angiography

CA was performed using Judkins method, via femoral approach, using cranial and caudal angles in the right and the left inclined planes at 30 fps. Patients’ CA images were analyzed by interventional cardiologists who were blinded to the study. CAE was defined by Falsetti and Carroll;^21^ our study used the same method. Normal segments were defined as the absence of stenosis or ectasia determined by CA. Cases of CAE with coronary stenosis were excluded from the study.

### Statistical analysis

Statistical analysis was carried out using SPSS v25 (SPSS Inc., USA). The normality of continuous variables was tested by the Kolmogorov-Smirnov test and presented as mean and standard deviation or median and interquartile range, according to data normality. Normally distributed continuous variables were compared by Student’s t test, and Mann-Whitney U test was used for non-normal distribution. Student’s t test for unpaired values was used. Categorical data were compared using chi-square test. In univariate linear regression analysis, variables with a significance level p < 0.25 were defined as potential risk markers and included as common variables in the whole variable model. Logistic regression analysis was performed to obtain independent determinants of CAE. A 2-sided p < 0.05 was statistically considered significant.

## Results

Of 226 patients, 8 were excluded because of myocardial infarction and left ventricular dysfunction; 5 were excluded due to left ventricular hypertrophy and heart valve disease, and 6 were excluded due to HT and renal failure (n = 6). In addition, 3 patients were excluded because of other reasons such as cerebrovascular disease, liver dysfunction, autoimmune disease, neoplastic disease, and osteoporosis (n = 3). After these exclusions, 204 patients were enrolled. One hundred two patients with isolated CAE and no coronary artery stenosis (56 men and 46 women; mean age 60.4 ± 8.8 years) were enrolled as patient group, and the control group consisted of the same number of consecutive subjects with angiographically normal coronary arteries (55 men and 47 women; mean age 61.2 ± 9.1 years).

Patients’ data are shown in Table 1 . The demographic characteristics showed age and sex matched groups. CAD risk factors such as DM were also similar, but other risk factors (smoking, HT, HL, and family history) were significantly higher in the CAE group than in the control group (p < 0.001, p < 0.001, p = 0.006, and p = 0.022, respectively). No changes were observed in terms of treatment regimens.

**Table 1:** – Baseline characteristics of the study population

Variables	Patients with CAE (n = 102)	Control group (n = 102)	P value
Age, years	60.4 ± 8.8	61.2 ± 9.1	0.422
Sex (male), n, (% )	56 (54.9)	55 (53.9)	0.740
BMI (kg/m^2^)	27.4±3.1	25.2± 3.2	0.317
Diabetes mellitus, n (%)	15 (14.7)	12 (11.7)	0.422
Smoking, n, (%)	40 (39.2)	18 (17.6)	< 0.001
Hypertension, n (%)	38 (37.2)	27 (26.4)	< 0.001
Hyperlipidemia, n (%)	22 (21.5 )	10 (9.8)	0.006
Family history, n, (%)	15 (14.7)	8 (7.8)	0.022
**Previous medications, n, (%)**			
Acetylsalicylic acid	20 (19.6)	16 (15.6)	0.224
Betablockers	22 (21.5)	17 (16.6)	0.314
ACEI/ARB	17 (16.6)	13 (12.7)	0.509
Statins	10 (9.8)	7 (7.1)	0.356
LVEF, (%)	60.2±3.4	61.3±3.9	0.533
SBP (mmHg)	122.0±9.1	118.6±7.5	0.424
DBP (mmHg)	88.0±7.2	85.6±4.1	0.358
Heart rate (beat/m)	75.2±9	73.8±7	0.411
Hemoglobin, g/dL	13.1+1.8	12.7+1.7	0.388
White blood cell, 10^3^/mL	8.6±2.9	8.4±2.2	0.758
Platelet count, 10^3^/mL	232.7±77.4	244.8±75.2	0.554
FPG (mg/dL)	127.1±42.8	123.1±45.2	0.146
Creatinine, mg/dL	0.86 (0.75-0.99)	0.85 (0.75-0.97)	0.785
eGFR, mL/min	92.3 (68.9-105.6)	93.8 (74.9-107.9)	0.656
Total cholesterol, mg/dL	168.0±36.0	158.2±39.8	0.411
HDL (mg/dL)	33.6±7.0	39.8±8.0	0.012
LDL (mg/dL)	123.2±32.6	98.4±29.4	< 0.001
Triglyceride, mg/dL	129 + 61	122 + 58	0.188
C-reactive protein, mg/dL	1.25 (0.50-2.84)	0.62 (0.27-1.16)	< 0.001
Albumin, g/dL	3.76 + 0.42	4.02 + 0.32	< 0.001
CAR, *100	32 (12-68)	16 (6-30)	< 0.001

*ACEI: angiotensin-converting enzyme inhibitor; ARB: angiotensin receptor blocker; BMI: body mass index; CAR: C-reactive protein to albumin ratio; DBP: diastolic blood pressure; eGFR: estimated glomerular filtration rate; FPG: fasting plasma glucose; HDL: high density lipoprotein; LDL: low density lipoprotein; LVEF: left ventricular ejection fraction; SBP: systolic blood pressure*

Assessment of BMI, SBP, DBP, LVEF, heart rate, and fasting plasma glucose did not show any significant differences. Lipid panel parameters, triglycerides, and total cholesterol groups were similar; HDL was higher in the controls (p = 0.012), and LDL was higher in patients with CAE (p < 0.001). CRP, albumin, and the CAR differed significantly between the groups (p < 0.001). There was a similarity between the groups in terms of other laboratory parameters.

Applying a univariate logistic regression model, DM, smoking, HT, and the CAR correlated with CAE. Regression analysis revealed that smoking, HT, and the CAR were independent predictors of CAE (smoking: OR 1.812 [95% CI 1.124 – 2.655; p = 0.024], HT: OR 2.175 [95% CI 1.156 – 4.227; p < 0.001], CAR: OR 2.202 [95% CI 1.184 – 5.365; p < 0.001]) ( Table 2 ).

**Table 2:** – Factors associated with coronary artery ectasia

Linear regression analysis				Logistic regression analysis		
	Coefficients	95% CI	P value	OR	95% CI	P value
Age, years	0.052	0.013-0.107				
LVEF	0.002	−0.018-0.026				
BMI (kg/m^2^)	0.030	0.010-0.073				
Diabetes mellitus	0.168	0.011- 0.524	0.024*	1.277	0.811-1.613	0.102
Smoking	0.322	0.010-1.114	0.007*	1.812	1.124-2.655	0.024*
Hypertension	0.533	0.017-1.010	0.003*	2.175	1.156-4.227	< 0.001*
Hyperlipidemia	0.025	−0.020-0.056				
SBP (mmHg)	0.068	−0.017-0.122				
DBP (mmHg)	0.024	−0.002-0.048				
Heart rate (beat/m)	0.074	−0.024-0.172				
CAR, *100	0.618	0.119-1.496	< 0.001*	2.202	1.184-5.365	< 0.001*
HDL (mg/dL)	−0.076	−0.312-0.025				
LDL (mg/dL)	0.009	−0.057-0.020				

*P value < 0.05. Variables with p < 0.25 in univariate regression were included into multivariate regression. BMI: body mass index; CAR: C-reactive protein to albumin ratio; DBP: diastolic blood pressure; HDL: high density lipoprotein; LDL: low density lipoprotein; LVEF: Left ventricular ejection fraction; SBP: systolic blood pressure.

## Discussion

This reports shows that the CAR was significantly higher in the CAE group than in the control group. To our knowledge, we are the first to show that the CAR is closely associated with CAE.

It is not clear which local or global factors are involved in CAE pathogenesis. It is reported that CAE is caused by the widespread abnormality in the vascular wall holding multiple segments and that it represents saccular ectasia rather than fusiform ectasia.^22^ A relatively limited study on prognosis was performed for patients with CAE. Thirty years ago, the largest cohort study of CAE found that aneurysmal patients had a 5-year mortality rate of 26%.^23^ Kajinami et al.^24^ examined the autopsy of a patient with CAE and familial hypercholesterolemia, who died in the twentieth century due to acute myocardial infarction. Microscopic examination revealed a large amount of plasma cells, macrophages, and lymphocyte infiltration in the intimal/medial layers of the coronary arteries. Evidence of atherosclerotic reactions such as typical common hyalinization, focal calcification and fibrosis, lipid accumulation, intimal and medial damage, cholesterol, hemorrhage, and foreign body giant cell were observed during pathological examination of CAE.

Another potential factor leading to the development of CAE is nitric oxide (NO), which may cause coronary dilatation due to over-stimulation of the endothelium. Many patients have been given chronic glyceryl trinitrate for angina that may worsen ectasia via NO stimulation. These patients may have CAD, and atherosclerosis has been shown to cause inappropriate release of endothelial NO.^25^ Quyyumi et al.^26^ demonstrated the relationship between NO and atherosclerosis and reported that coronary vascular dilatation was caused by increased NO due to acetylcholine without angiographically proven atherosclerosis.

The underlying pathological mechanism of CAE is still not fully understood. Although a definite relationship between atherosclerosis and CAE has not been confirmed, CAE is considered to be a variant of CAD and the main cause of CAE is atherosclerosis.^23 , 27 - 29^ The role of inflammation in the process of atherosclerosis is well known.^28 - 30^ Atherosclerosis is associated with aneurysm formation that extends to tunica media during an inflammatory process, which eventually ends with degeneration of the cystic media.^31^ Previous studies have shown that inflammatory markers such as plasma soluble adhesion molecules, leukocytes, adiponectin, lipoprotein-associated phospholipase-A2, CRP, plasminogen activator inhibitor-1, IL-1, TNF-alpha, and IL-10 have been significantly increased in patients with CAE.^32^

Many previous studies have shown that the CAR is associated with atherosclerosis and suggested that it should be considered as a marker of cardiovascular risk. This study found that the CAR was significantly higher in patients with CAE than in the control group, and it supports the hypothesis that atherosclerosis causes CAE.

Damaged ischemic or necrotic cells cause a systemic inflammatory response by releasing pro-inflammatory agents in tissue and plasma. The prognosis of the disease can change with the speed of inflammation.^33^ Atherosclerosis has been shown to be strongly correlated with increased serum CRP.^34^ In addition, CRP has been shown to be associated with endothelial dependent/independent coronary dysfunction in patients with CAD,^35^ suggesting that increased CRP may predict dysfunction in STEMI patients and may be a strong predictor of no-reflow phenomenon.^36^ In our study, elevated CRP levels showed a strong association between CAE and CRP.

Hypoalbuminemia is not only a risk factor; it also indicates poor prognosis in patients with STEMI.^37 , 38^ Increased inflammation has been documented to contribute to albumin synthesis and breakdown.^39^ Hypoalbuminemia leads to many complications including endothelial dysfunction as well as platelet aggregation and coronary artery stenosis induced by platelet dysfunction.^40 - 41^ In a study of 1,303 subjects with acute coronary syndrome, serum albumin levels were shown to be associated with severity of CAD.^10^ In our study, there was a negative correlation between serum albumin level and CAE.

It is believed that the CAR, as originally described by Fairclough et al.,^42^ is better than CRP and albumin alone for prediction of medical complications.^42^ Inflammation is one of the main characteristics of atherogenesis, and the CAR and demonstrates inflammatory conditions.

The CAR has recently been investigated as a potential biomarker for predicting the consequences of adverse cardiovascular events.^43^ Cagdas et al.^44^ showed that the CAR and the severity of CAD were associated. In malignant cancer patients, the CAR predicted prognosis and disease progression.^45 , 46^ Therefore, the CAR is a more reliable biomarker for prediction of disease severity. Previous reports that evaluated the CAR in CAD showed promising outcomes. A study of STEMI showed that white blood cell count, neutrophil to lymphocyte ratio, and the CAR correlated with the no-reflow phenomenon.^43^

The results of our study showed that the association of the CAR with CAE was significant. This is the first study to show an association between CAE and higher CAR levels. Increased CAR was a prognostic marker of CAE. The results of a study on the relationship between familial hypercholesterolemia and CAE showed that dyslipidemia was one of the causes of CAE.^47^ In our study, high LDL and low HDL levels were observed in patients with CAE. No significant change was found in triglyceride levels in patients with CAE compared to controls. A strong relationship has been shown between HT and CAE.^48^ In our study, the prevalence of HT was higher in patients with CAE, and HT was independently associated with CAE.

We observed that the CAR was higher in patients than in control group. We assume that higher CAR may predict the risk of atherosclerosis in patients with CAE. A review of the literature shows that CAE is not an innocent clinical condition and that larger studies are needed in the future to create the best strategy for treatment and risk management.

### Study limitations

More comprehensive and multicenter studies are needed to better explain the variability of inflammatory markers and the predictive role of serum CAR levels. The prognostic significance of the CAR was not evaluated, and it should be established in future investigations. This was a case-control study, and we were thus unable to obtain mortality data. Although the CAR is accepted as a new, sensitive myocardial marker, its specificity in determining the presence of CAE has been questioned, because many other conditions, especially other infections, may also increase CAR levels. CAR levels may be affected by other important factors, such as age, sex, and race. Finally, instead of using quantitative methods such as intravascular ultrasound, visual assessment was the only method used to diagnose and exclude patients.

## Conclusion

Our study shows that CAR levels are higher in patients with CAE compared to subjects with normal coronary arteries. The high levels of CAR may support the hypothesis that the CAR could be related to the development of CAE. In our study, high CAR levels were significantly correlated with CAE.
